# Cutaneous Metastases from Primary Liver Cancers: The Need for Knowledge and Differential Diagnosis

**DOI:** 10.3390/life11060559

**Published:** 2021-06-14

**Authors:** Gerardo Cazzato, Anna Colagrande, Antonietta Cimmino, Aurora De Marco, Paolo Romita, Caterina Foti, Leonardo Resta, Giuseppe Ingravallo

**Affiliations:** 1 Section of Pathology, Department of Emergency and Organ Transplantation (DETO), University of Bari “Aldo Moro”, 70124 Bari, Italy; anna.colagrande@gmail.com (A.C.); micasucci@inwind.it (A.C.); leonardo.resta@uniba.it (L.R.); 2 Section of Dermatology, Department of Biomedical Sciences and Human Oncology, University of Bari “Aldo Moro”, 70124 Bari, Italy; aurorademarco94@gmail.com (A.D.M.); romitapaolo@gmail.com (P.R.); caterina.foti@uniba.it (C.F.)

**Keywords:** skin metastasis, hepatocarcinoma, cholangiocarcinoma, metastatic, cancer, dermopathology

## Abstract

Primary skin tumors are certainly more frequent than metastatic tumors, but the latter can sometimes be the first sign of otherwise unrecognized neoplastic pathology and always correspond to an advanced stage of the disease. Among the various neoplasms that can metastasize in cutaneous districts, skin metastases from primary malignant neoplasms from the liver and biliary tract are infrequent, and when they do occur they can pose differential diagnosis problems to the pathologist. Here we present two cases of metastatic skin lesions, respectively originating from the liver and the intrahepatic biliary tract, and we conduct a brief review of the current literature.

## 1. Introduction

The skin can be the site of primary, benign or malignant neoplasia, but also of metastases originating from neoplasms of other parts of the body. Although primary skin cancers are more frequent [1], cutaneous metastases develop in 4.5% of all patients with visceral malignant neoplasms [2]. Neoplasms of different districts may secondarily affect the skin with a decreasing incidence starting from breast, colon and melanoma in females and lung, colon and melanoma in males [3]. The histological diagnosis of skin metastases is quite simple in the case of well/moderately differentiated lesions, but it becomes difficult and challenging in the case of poorly differentiated neoplasms [3,4]. 

Primary liver cancer is globally the sixth most frequent cancer and the fourth leading cause of death from cancer. These cancers sometimes can metastasize to the skin; indeed, Hepatocarcinoma (HCC) metastasizes with a frequency ranging from 2.7% to 3.4% of all HCCs [5], and it may be the first sign of an underlying malignancy, with disease stages usually found to be advanced [6]. On the other hand, cholangiocarcinoma is a diverse group of epithelial cancers united by late diagnosis and poor outcomes [7]; cases of skin metastases of cholangiocarcinoma are extremely rare and, to date, about 35 cases have been reported in the literature [8].

Here, we present two cases of HCC skin metastases and intrahepatic cholangiocarcinoma occurring in our clinical–pathological practice and conduct a review of the literature discussing similarities and differences in previously reported cases. Finally, we focus on the most important clues for a correct differential diagnosis between metastatic cutaneous neoplasms and primary cutaneous neoplasms.

## 2. Cases Presentation

### 2.1. Case Number 1

**Figure 1:**
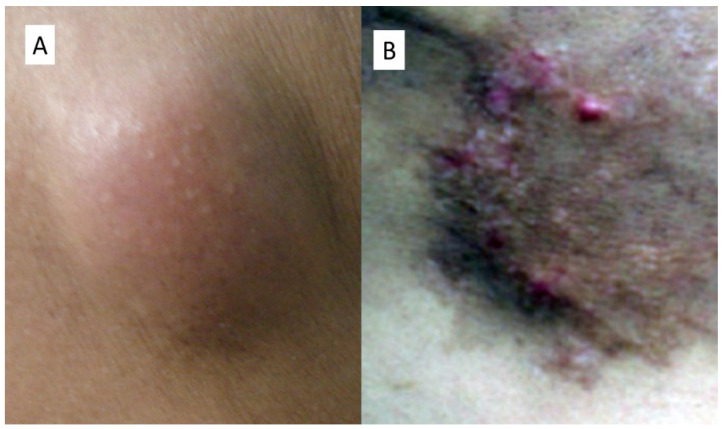
Clinical appearance of nodular metastasis of HCC of Case Number 1 (**A**) and of the localization in the periumbilical region of the Case Number 2 (**B**).

A 65-year-old man showed up in 2019 at the U.O.C. of Internal Medicine for worsening of clinical conditions related to a previous diagnosis of chronic HCV-related hepatitis. Following the execution of laboratory investigations that highlighted a dizzying rise in alpha-fetoprotein (αFP) equal to 703.7 ng/mL, an ultrasound-guided liver biopsy was conducted (in correspondence of VII liver segment) that concluded, histologically, for moderately differentiated hepatocellular carcinoma (Grade 3 according to the Edmonson score) associated with HCV-related end-stage cirrhosis. The patient was treated with radiofrequency thermalablation, and a year later he developed a new primary hepatic neoplastic lesion of the VIII segment. Therefore, he underwent thermoablation again. In January 2021, during routine checks, the patient underwent a total body CT scan following the finding of a nodule in the skin of the right thorax that was solid, vascularized, about 4 cm in maximum diameter, and slightly erythematous in relation to the surrounding skin (Figure 1). The patient underwent surgery and the sample, fixed in 10% neutral buffered formaldehyde, was sent to our section of Pathological Anatomy. 

### 2.2. Case Number 2

A 73-year-old man had been diagnosed five years earlier with intrahepatic cholangiocarcinoma, for which he had undergone a partial hepatectomy. A few years later he was sent to the Plastic Surgery department for the appearance of a swelling in the paraumbilical area. Following a medical examination, they opted for surgery, which, once performed, showed suspicious repetitions, some of which surfaced in the stomach wall and, also, in the skin. This lesion was a plaque in the periumbilical region of brownish color, with small tokens that seem to extend from the skin plane. 

## 3. Material and Methods

Both cutaneous samples were sampled according to the guidelines, and obtained sections were exposed to processing. After inclusion in paraffin and microtome cutting, 5 micron (µm) thick sections were obtained, stained with routine staining (Hematoxylin-Eosin) and observed under an optical microscope. As usual, many pertinent antibodies were used for immunohistochemistry. Informed consent from both patients was obtained for enrollment in the present study. In addition, a brief review of current literature was conducted using Pubmed and Web of Science (WoS) as search engines with the words “Skin metastasis” OR “Cutaneous metastasis” OR “Metastatic HCC” and “Hepatocarcinoma” OR “Cholangiocarcinoma”.

## 4. Results

### 4.1. Case Number 1

**Figure 2:**
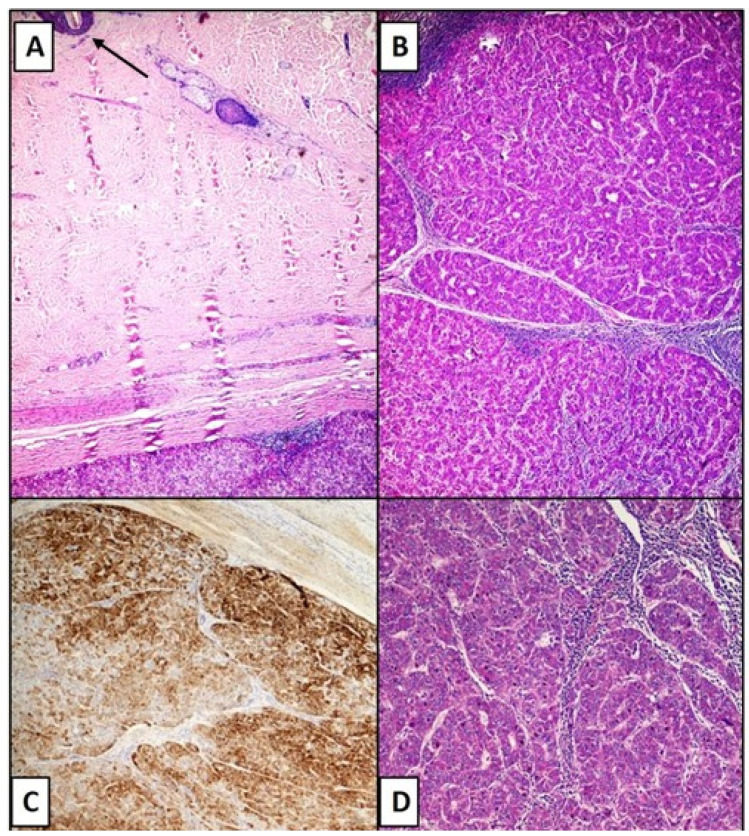
(**A**) Photomicrograph showing the distance between the epidermis (see top left) and the point of metastasis of the HCC (see below) (Hematoxylin-Eosin, 10×). (**B**) Bundles and cords of hepatocyte-like cells densely packed and interspersed with small blood vessels resembling hepatic sinusoids (Hematoxylin-Eosin, 20×). (**C**) Immunostaining for HepPar-1 strongly positive in the neoplastic component. Note the cytoplasmic, granular positivity (HepPar-1 immunolabel, 20×). (**D**) Details of microphotograph B (Hematoxylin-Eosin, 40×).

The histological sections showed the presence of a nodular lesion, with regular contours, mainly localized at the dermo–hypodermic junction (Figure 1), consisting of bundles of tumor cells that resembled hepatocytes, with a different trabecular and cordon disposition (Figure 2B,D). The stroma was composed of vascular spaces lined with a single layer of endothelial cells (Figure 2D). These spaces had varying degrees of expansion and were positive for the immunohistochemical reaction for CD34 (not shown). The immunohistochemical reaction for Hepar-1 was strongly positive, with granular, cytoplasmic positivity (Figure 2C). Reaction for Desmin, Vimentin, Melan-A, HMB-45, SOX-10, EMA, and Smooth muscle actin were all negative.

### 4.2. Case Number 2

**Figure 3:**
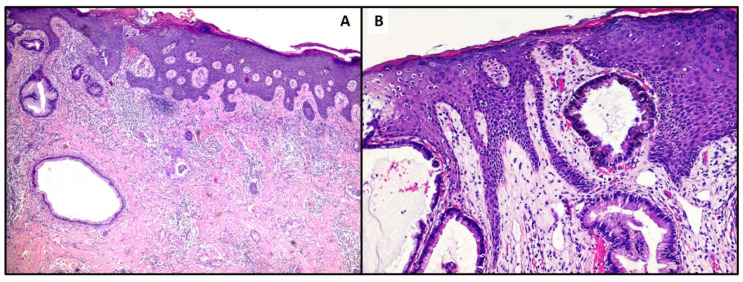
(**A**,**B**). Photomicrograph showing superficial dermis and epidermis colonization by gland-like structures. The latter are constituted of cells with round to oval nuclei, high Nucleus/Cytoplasm ratio and cytological atypia (Hematoxylin-Eosin, 20× and 40×).

The histological sections of this case revealed the presence of atypical glands consisting of cuboidal to columnar neoplastic elements, with pale cytoplasm, and sometimes eosinophilic, round to oval nuclei, with the presence of mucin secretion. (Figure 3A). Curiously, some glandular elements were related to the epidermal ridges (Figure 3B). Immunostains for Cytokeratin 7 (CK-7) were strongly positive, with a focal positivity for CDX-2. Conversely, the reactions for CK-20 and TTF-1 were negative. The histological and immunophenotypic picture was oriented towards the biliopancreatic origin of the metastatic skin lesion.

## 5. Discussion

Cutaneous metastasis of hepatocellular carcinoma is infrequent, accounting for less than 0.8% of all known cutaneous metastases and occurring in 2.7–3.4% of HCCs [6]. The most common sites of cutaneous spread of HCC are the face, scalp, chest, and abdomen [6,7]. Clinically, these lesions can simulate different types of skin diseases such as pyogenic granuloma [9] rather than angioma [10] or granuloma teleangiectaticum [11]. In the literature, they have been described as dissemination of a primitive HCC, although Queen et Al. reported four cases of HCC metastases after liver transplantation [6]. In the vast majority of the works, patients with HCC skin metastases had already had a previous diagnosis of hepatic malignancy, but there are some reports of cases in which skin presentation was the first sign of HCC. For example, Patel et al. [12] described a case of an 83-year-old man in whom the first clinical presentation of HCC was at the level of the left eyebrow, and only later did he develop ascites and anasarca as a result of advanced liver disease. Paolino et al. conducted a systematic review of cases of neoplastic alopecia attributable to metastases from malignant neoplasms in the scalp [13].

In addition, Amador et al. [14] described a case of an 80-year-old patient with an abscess at the level of the shoulder, who only after this skin manifestation was framed for a neoplastic pathology originating from the liver. In our case, however, the primary hepatic pathology was well known. The clinical presentation at the thorax level nevertheless posed diagnostic dilemmas from a clinical point of view, because although the possibility of determining subcutaneous neoplastic implants after surgery for HCC is described in the peri-surgical site, this anatomic location was far enough from the surgical access site previously practiced to obtain a liver biopsy. From a histological point of view, the recognition of a cutaneous metastasis of HCC requires not only an evaluation of the morphology. In fact, although the metastasis may have histological characteristics that lead to suspicion (bundles of hepatocyte-like cells with a solid-trabecular pattern, with pleomorphic nuclei and evident nucleoli), immunohistochemical investigations constitute a fundamental step to disentangle the various differential diagnoses. Among these, Hepar-1 constitutes a reference point, with a sensitivity and specificity index that is 82% to 95% [5], but in recent years Arginase-1 (Arg-1) and Glypicane-3 (Gly-3+) have also acquired a certain role [6]. Isa et al. [5] describe in detail that to be considered effectively positive, the immunohistochemical staining must be strong, cytoplasmic, and granular in spite of a small part of malignant neoplasms that can focal, in an aberrant sense, express it. Briefly, all this serves to allow a correct differential diagnosis with respect to primary malignant skin neoplasms (such as squamous cell carcinoma, melanoma and adnexal cutaneous neoplasm) and secondary non-hepatic skin neoplasms originating from organs such as lung, gastrointestinal tract, kidney, bladder, prostate, adrenal carcinoma and/or pancreatic carcinoids [5,6,14,15,16,17,18,19]. Hu SC et al. [18] provide their experience related to a medical center in Taiwan in which they describe only 4 cases of skin metastases out of 1189 cases of HCC, equal to 0.34% of cases. In their experience, metastases originated more frequently from the lung (2.42% and 1.8% of cases, respectively), with the liver represented in the middle position as a frequency rate. 

Skin metastases from cholangiocarcinoma are very rare occurrences, and in a recent review, Liu M. et al. [8] described about 30 cases published in the literature. The authors’ analysis shows that the median age of development of cutaneous metastases from cholangiocarcinoma was about 60 years, with a male/female ratio of 3.29, and in 8 cases of the 30 examined, the skin manifestations were the first sign of cholangiocarcinoma. Skin lesions tend to present as nodules, papules, erythema and lesions with or without ulcers, which were sized from 0.3 cm to 4 cm; mostly they present on scalp (36.7% of all patients) [8,20] although other anatomical locations such as the sternum region were described by Fernández López AJ et al. [21] or thigh by Varma K. et al [22]. In our case, we believe that they are real neoplastic implants due to previous surgery; in fact, unlike our first case presented, the relationship of anatomical proximity suggests this hypothesis may be more likely. Morphologically, the routine stains allow us to hypothesize in which field of lesions we move (as, for example, in our case), but certainly a thorough knowledge of the clinical history and any intercurrent pathologies must be pursued. From an immunohistochemical point of view, it is essential to evaluate the positivity of Cytokeratin 7 and at the same time the negativity of Cytokeratin 20, data that allow orienting the diagnosis towards the probable biliopancreatic origin [23], always in the presence of a consolidated remote anamnesis in this sense. Furthermore, the positivity for CDX-2 adds a further clue, not forgetting that appropriate additional immunostaining is absolutely necessary to exclude other lesions (for example, TTF-1 for pulmonary and/or thyroid origin). 

## 6. Conclusions

We have reported two cases of cutaneous metastases from HCC and intrahepatic cholangiocarcinoma, accompanying them with divergent morphological and immunophenotypic characteristics and trying to make a comparison with the cases already described. New and larger case series, together with molecular studies, will be needed to investigate the intricate mechanisms through which the skin becomes occasionally host to a variety of malignant neoplasms.
